# Lymphadenectomy and adjuvant therapy in endometrial carcinoma: role of adjuvant chemotherapy

**DOI:** 10.1038/sj.bjc.6600468

**Published:** 2002-08-12

**Authors:** I Otsuka, T Kubota, T Aso

**Affiliations:** Department of Obstetrics and Gynecology, Tokyo Medical and Dental University Hospital 1-5-45 Yushima, Bunkyo-ku, Tokyo 113-8519, Japan

**Keywords:** endometrial carcinoma, lymphadenectomy, adjuvant therapy, chemotherapy, recurrence

## Abstract

To evaluate the therapeutic benefit of lymphadenectomy and adjuvant therapy, in particular chemotherapy, we retrospectively analysed survival rates and patterns of recurrence of endometrioid adenocarcinoma in 106 patients who underwent surgery including retroperitoneal lymphadenectomy. Adjuvant chemotherapy was administered to 46 patients (42 received a platinum-based regimen) and pelvic irradiation to 12. The 5-year survival rate of 23 patients with lymph node metastasis was worse than that of patients without lymph node metastasis (60% *vs* 96%, *P*<0.0001). Recurrence was observed in 14 patients (10 patients with chemotherapy, two with irradiation, and two without adjuvant therapy); the first site of recurrence was in distant sites in 12 patients; recurrence in the pelvic sidewall or exclusively in lymph nodes was not observed. The 5-year survival rate of 18 patients with lymph node metastasis treated with chemotherapy, was 61% including all 14 with macroscopically positive nodes and all nine with paraaortic metastasis. Of seven patients with bulky positives nodes, three patients with bulky paraaortic nodes died of the disease, three of the four patients with bulky pelvic but without bulky paraaortic nodes had no recurrence. In summary, lymphadenectomy may afford a survival benefit via the debulking of macroscopically positive nodes, and the predominance of distant recurrences suggests that chemotherapy is a suitable choice as an adjuvant therapy in endometrial carcinoma after lymphadenectomy.

*British Journal of Cancer* (2002) **87**, 377–380. doi:10.1038/sj.bjc.6600468 www.bjcancer.com

© 2002 Cancer Research UK

## 

In endometrial carcinoma, lymph node metastasis is one of the most important prognostic factors for disease recurrence and death (Disaia *et al*, 1985; Lurain *et al*, 1991; Morrow *et al*, 1991); however, the role of lymphadenectomy in the management of endometrial carcinoma still remains controversial. Lymphadenectomy is recommended for predicting patients prognosis and for tailoring postoperative therapy in many studies (Chuang *et al*, 1995; Kilgore *et al*, 1995; Blythe *et al*, 1997; Yokoyama *et al*, 1997). However, Creutzberg *et al* (2000) stated that lymphadenectomy cannot be considered a standard procedure because its benefit is unclear. Since lymphadenectomy is performed mainly with diagnostic intent, positive lymph nodes are often left unresected (Creasman *et al*, 1987); therefore the therapeutic benefit of lymphadenectomy, which should be evaluated by resection of macroscopically positive nodes as well as microscopically positive nodes, is still unclear.

The majority of patients at a risk of recurrence receive adjuvant radiotherapy following surgery. However, although pelvic radiotherapy decreases vaginal stump recurrence it does not improve survival (Aalders *et al*, 1980; Creutzberg *et al*, 2000), since many patients develop distant recurrences. Recently, in some studies, adjuvant chemotherapy has been administered to patients at a risk of recurrence (Yokoyama *et al*, 1997; Larson *et al*, 1998).

We have been performing lymphadenectomy that removes negative and positive nodes including macroscopically enlarged nodes with therapeutic intent on endometrial carcinoma patients. In our institution, moreover, adjuvant chemotherapy has been performed on patients at a high risk of recurrence. In this retrospective study, our objectives are to evaluate the therapeutic benefit of lymphadenectomy and adjuvant therapy, in particular chemotherapy, by analysing survival rates and patterns of recurrences.

## PATIENTS AND METHODS

Between 1980 and 1999, 143 women with endometrial adenocarcinoma were treated at the Department of Obstetrics and Gynecology, Tokyo Medical and Dental University Hospital. Excluding patients with concomitant malignancy (eight patients with ovarian carcinoma, one with fallopian tube carcinoma) and histology other than endometrioid tumour (four patients), 130 patients had stage I–IV endometrioid adenocarcinoma. Of these patients, nine had abdominal or pulmonary spread, and 15 with stage I–III disease did not undergo lymphadenectomy. Thus, 106 patients who underwent surgery including lymphadenectomy were studied.

All 106 patients underwent abdominal hysterectomy, bilateral salpingo-oophorectomy, and pelvic lymphadenectomy. Systematic pelvic lymphadenectomy is performed to remove the following lymph nodes with surrounding fat pads: common iliac, external iliac, internal iliac, obturator, and deep inguinal nodes. Before 1988, paraaortic nodes were dissected in patients with gross paraaortic node swelling, and since 1988 paraaortic nodes have been dissected in the patients at risk for paraaortic node metastasis such as those with grossly positive pelvic and/or paraaortic nodes, gross adnexal metastasis, high grade tumours, and deep myometrial invasion which is determined intraoperatively by gross inspection of a sectioned uterine corpus. Paraaortic nodes are systematically dissected as follows: through the incision of the posterior peritoneum at the small bowel mesentery, lymph nodes anterior and lateral to the aorta and inferior vena cava are removed up to the renal vessel. The total number of patients in our study who had paraaortic node dissection was 47 (44%). Even when grossly positive nodes were found, complete node dissection was performed in those areas. The mean number of nodes removed was 19 (range, 2–43) for the pelvic nodes and 7 (range, 1–24) for the paraaortic nodes. None of the patients had gross residual disease after surgery.

All patients had endometrioid adenocarcinomas with or without squamous differentiation: 67 patients (63%) with grade 1, 27 (25%) with grade 2, and 12 (11%) with grade 3 tumours. The stages, which were defined according to the 1989 International Federation of Gynecology and Obstetrics staging system, of the 106 patients were as follows: stage I, 66 patients (62%); stage II, 12 patients (11%); stage III, 27 patients (25%); and stage IV, 1 patient (1%). The stage IV patient had inguinal node metastasis with pelvic and paraaortic lymph node involvement, but did not have other distant metastases. Patients treated prior to 1989 were retrospectively staged based on their surgical-pathologic findings; the results of peritoneal cytology for 20 patients (19%) treated before 1988, in whom peritoneal washing was not obtained, was considered as negative. The patients ranged in age from 27–81 years (mean, 56 years).

Adjuvant therapy was administered to 58 patients (55%). The standard adjuvant therapy of our institution for the patients with risk factors for recurrence, namely, deep myometrial invasion, cervical extension, or extrauterine disease, has been chemotherapy. From 1980 to 1983, a combination of cyclophosphamide 200 mg body^−1^ and 5-fluorouracil 500 mg body^−1^ was the preferred regimen, which was administered to four patients. Since 1984, five cycles of platinum-based chemotherapy at a 4-week interval were administered in standard doses including cisplatin at 50 mg m^−2^, doxorubicin at 40–50 mg m^−2^ and cyclophosphamide at 400–500 mg m^−2^ (CAP); patients with impaired renal function received carboplatin instead of cisplatin. A platinum-based regimen was administered to 42 patients, including all patients with macroscopically positive nodes or paraaortic metastasis. Ten other patients in poor health or who refused chemotherapy were irradiated with a 50.6 Gy external beam to the whole pelvis; radiotherapy was delivered in a daily fraction of 2.2 Gy, four times a week. Two other patients were treated with a combination of pelvic radiotherapy and chemotherapy. None of the patients received preoperative radiotherapy.

Factors assessed were as follows: lymph node metastasis, size of positive lymph nodes, non-nodal extrauterine diseases (positive peritoneal washing, and adnexal, serosal, and vaginal involvement), sites of the first recurrence, and survival time. In this study, a microscopically positive lymph node was defined as a node with a size ⩽1 cm, macroscopically positive lymph node a node with a size >1 cm and a bulky positive node a node with a size >2 cm. Survival time was calculated from the date of surgery to the date of death or last contact. Survival times of the patients who died of causes other than endometrial carcinoma were censored at the date of death. Two patients, with stage IIIC disease, who died with disease status unknown (2, 19 months), were considered to have died of disease. The median follow-up was 68 months (range, 2–233 months), including death cases. Survival curves were estimated using the Kaplan–Meier product-limit method and compared using the log-rank test. The statistical analysis was performed using the StatView software (version 4.5; Abacus Concepts Inc., Berkeley, CA, USA).

## RESULTS

Retroperitoneal lymph node metastasis was found in 23 (22%) of 106 patients: 21 (20%) in the pelvic, and nine (8%) in the paraaortic region. Of the 21 patients with pelvic node metastasis, seven (33%) had coexisting paraaortic node metastasis, including one patient who had inguinal lymph node metastasis. Paraaortic node metastasis without pelvic node involvement was found in two patients.

The 5-year survival rate of patients with lymph node metastasis was 60%, which was significantly worse than that of the patients without lymph node metastasis, i.e., 96% (*P*<0.0001). In the 23 patients with lymph node metastasis, the 5-year survival rates of the patients with pelvic node metastasis alone and that of the patients with paraaortic node metastasis were 71% and 44%, respectively; the difference was not statistically significant (*P*=0.12).

Recurrence developed in 14 (13%) of 106 patients (Table 1

**Table 1:**
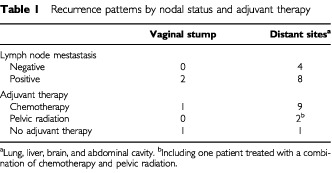
Recurrence patterns by nodal status and adjuvant therapy

). In node-negative patients, only distant recurrences developed (the lung in three, the liver in one; three stage IC patients and one stage IIIB patient); retroperitoneal lymph node recurrence was not observed. In node-positive patients, recurrences developed in distant sites in eight patients (the liver in three, the lung in two, the brain in two, the abdominal cavity in one), and in the vaginal stump in two. Lymph node recurrences, which were found in the paraaortic region, developed in two patients concomitantly with distant metastasis (the lungs in one, and the liver in the other), both of whom had macroscopically positive pelvic nodes. Recurrence exclusively in lymph nodes did not develop in any of the patients. No pelvic sidewall recurrence was observed. Vaginal stump recurrence developed in two patients, one treated with adjuvant chemotherapy and the other who did not receive adjuvant therapy; the former patient was alive without recurrence of the disease for more than eight years after surgical resection of the tumour and vaginal brachytherapy, but the latter patient who received vaginal brachytherapy developed a recurrence in the lower vagina followed by pulmonary metastases.

**Figure 1:**
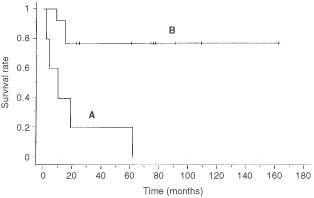
Survival of patients with lymph node metastasis: patients with either positive peritoneal washing, gross adnexal involvement, or serosal involvement (**A**) *vs* patients without these extrauterine diseases (**B**).

Of the 23 patients with lymph node metastasis, adjuvant chemotherapy was administered to 18, including all 14 with macroscopically positive nodes and all nine with paraaortic node metastasis; the 5-year survival rate of the 18 patients was 61%, and those of the patients with pelvic node metastasis alone and the patients with paraaortic node metastasis were 78% and 44%, respectively. Five patients with either positive peritoneal washing, gross adnexal, or serosal involvement had a significantly worse survival rate than 13 patients without these extrauterine diseases (20% *vs* 77%, *P*=0.0004, Figure 1

). Grade had no significant effect on survival (5-year survival, 83% for grade 1 *vs* 50% for grade 2 or 3).

All 14 patients with macroscopically positive nodes received a platinum-based chemotherapy (Table 2

**Table 2:**
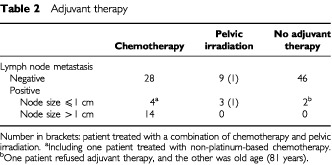
Adjuvant therapy

). The 5-year survival rate of patients with macroscopically positive nodes was not different from that of patients with microscopically positive nodes (60% *vs* 62%, respectively). Of the 14 patients, seven had bulky positive node(s), and their 5-year survival rate was 43%; whereas three patients who had bulky paraaortic nodes died of the disease, three of four patients with bulky pelvic but without bulky paraaortic nodes had no recurrence.

## DISCUSSION

This study shows that lymphadenectomy may afford a survival benefit via the debulking of macroscopic lymph node metastases. Moreover, the patterns of recurrence, that is, the predominance of distant recurrences, suggests that chemotherapy is a more suitable choice as an adjuvant therapy than radiotherapy in endometrial carcinoma after lymphadenectomy.

Lymphadenectomy appears to reduce lymph node recurrence in both patients with positive and negative nodes. Our observation that recurrences exclusively in lymph nodes did not develop in the patients with positive nodes appears to support the therapeutic benefit of lymphadenectomy. Moreover, in patients in whom lymph node metastasis was not detected, lymphadenectomy may have removed an unrecognised micrometastatic disease in lymph nodes. The majority of recurrences after lymphadenectomy developed in distant sites such as the lung, liver and brain; these recurrences may be caused by haematogenous spread that may already have occurred at the time of surgery or could not be prevented by dissection of the lymph nodes.

Also in patients with macroscopically positive nodes, lymphadenectomy may have a therapeutic benefit: the survival rate of these patients was not different from that of the patients with microscopically positive nodes. Even in patients with bulky positive nodes in the pelvic region, a favourable survival is expected by removal of these nodes and adjuvant chemotherapy.

However, certain subsets of node-positive patients have a poor survival: those with bulky positive paraaortic node(s). Patients with paraaortic node involvement have been reported to have a worse 5-year survival rate than those with pelvic node metastasis alone: 27–75% *vs* 67–100%, respectively (Potish *et al*, 1985; Corn *et al*, 1992; Hicks *et al*, 1993; Kadar *et al*, 1994; Onda *et al*, 1997; Yokoyama *et al*, 1997). In our previous study, patients with paraaortic node metastases were at risk of pulmonary metastases (Otsuka *et al*, 2002); thus, a haematogenous spread such as pulmonary metastases developing after paraaortic node involvement may reduce the survival.

Node-positive patients with either positive peritoneal cytology, gross adnexal, or serosal involvement also have a poor survival. This type of disease spread, namely the coexistence of lymph node metastasis and non-nodal extrauterine diseases, may indicate a widely metastasised disease; in these patients lymphadenectomy may have a limited value. In contrast, if node-positive patients do not have these non-nodal extrauterine diseases, which appears to mean that the disease is limited to the lymphatic system, lymphadenectomy may have a therapeutic value in combination with an adjuvant therapy.

Considering that the majority of recurrences develop in distant sites, chemotherapy appears to be a more suitable choice as an adjuvant therapy than radiotherapy in patients who underwent lymphadenectomy. Adjuvant pelvic irradiation following surgery without lymphadenectomy only reduced the rate of vaginal stump recurrence, but did not improve the survival rate (Aalders *et al*, 1980; Creutzberg *et al*, 2000); in patients who underwent this treatment, distant metastases developing from undetected and untreated paraaortic nodes that were located outside of the irradiation field may decrease their survival; paraaortic node metastases were found in 32–78% of the patients with pelvic node involvement (Chen *et al*, 1985; Morrow *et al*, 1991; Yokoyama *et al*, 1997; Onda *et al*, 1997; Hirahatake *et al*, 1997) and in 16–44% of the patients with deep (>½) myometrial invasion (Morrow *et al*, 1991; Yokoyama *et al*, 1997; Hirahatake *et al*, 1997).

Extended-field irradiation that consists of pelvic and paraaortic irradiation, or whole abdominal irradiation after surgery including paraaortic with or without pelvic node sampling appears to have a therapeutic effect on patients with positive paraaortic nodes. However, these types of radiotherapy do not appear to be effective for cases of distant metastases, which are the majority of recurrences of endometrial carcinoma. Also, these types of irradiation frequently cause bowel complication especially after dissection of the paraaortic nodes (Komaki *et al*, 1983; Rose *et al*, 1992; Hicks *et al*, 1993).

It has not been proved that irradiation can eradicate tumour cells in macroscopically enlarged lymph nodes. In contrast, surgical resection of macroscopically enlarged nodes is presumed to have a debulking effect. In cervical carcinoma, patients with macroscopic lymph node metastasis and who have a poorer survival rate than patients with only microscopic lymph node metastasis (Monk *et al*, 1994) had improved outcomes by debulking of enlarged positive lymph nodes (Cosin *et al*, 1998). Our study, like the study of Cosin *et al*, demonstrated similar survivals for patients with completely resected lymph nodes, whether they were microscopically or macroscopically positive. In patients treated by adjuvant chemotherapy alone, pelvic recurrence is common after lymph node sampling or in the absence of lymphadenectomy (Mundt *et al*, 2001); in contrast, no pelvic sidewall recurrence was observed in our study, which included patients with bulky positive nodes. This suggests that after lymphadenectomy, irradiation to the whole pelvis may not be required.

Chemotherapy has been given as the standard adjuvant therapy in our institution; in endometrial carcinoma, response rates ranging from 38 to 76% have been reported with regimens of cisplatin, doxorubicin, with or without cyclophosphamide (Muss, 1994). In the present study, 5-year survival rates of chemotherapy-treated patients with pelvic node metastasis alone and those with paraaortic node metastasis were 78 and 44%, respectively. These survival rates favourably compare with reported survival rates of patients treated with adjuvant radiotherapy; 67–82% in patients with pelvic node metastasis alone and 27–58% in those with paraaortic node metastasis (Potish *et al*, 1985; Corn *et al*, 1992; Hicks *et al*, 1993; Kadar *et al*, 1994; Blythe *et al*, 1997).

We believe that pelvic lymphadenectomy should be performed in all patients with endometrial carcinoma, since approximately 20% of patients have pelvic lymph node metastasis (Hirahatake *et al*, 1997), including those with superficial invasion and low grade tumours. In contrast, paraaortic lymph nodes that were positive in approximately 10% of patients should be dissected only in patients at a high risk of metastases; Morrow *et al* (1991) noted that 98% of paraaortic node metastases were found in patients with either grossly positive pelvic nodes, grossly positive adnexal metastases, or outer one third myometrial invasion. In the present study, all patients with paraaortic node metastases had deep (>½) myometrial invasion.

The results of the present study show the therapeutic benefit of lymphadenectomy and adjuvant chemotherapy. However, distant recurrences may still develop in certain subsets of node-positive and node-negative patients. Further investigation to develop more potent systemic adjuvant treatments is needed.
